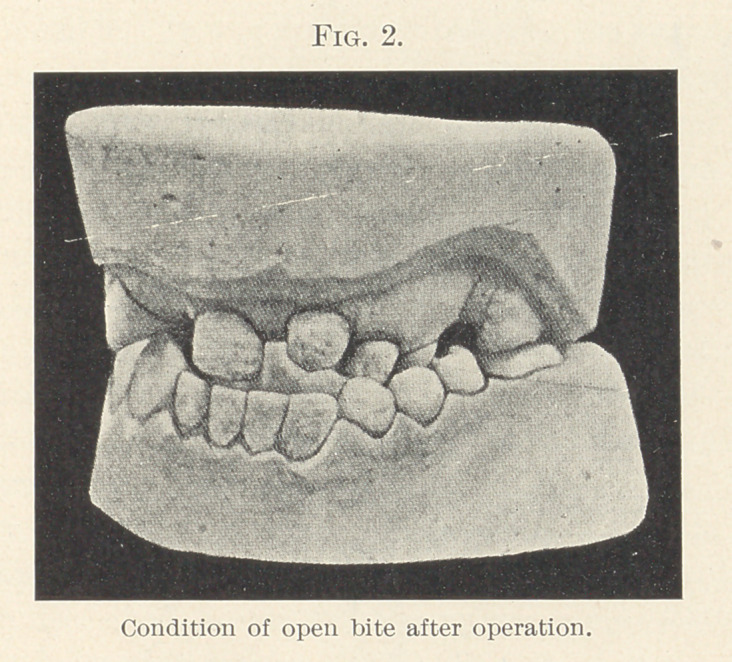# Reviews of Dental Literature

**Published:** 1903-04

**Authors:** 


Dental Use of Homœopathic Remedies.[1]—The author states that these remedies have given him excellent results in many cases, and he strongly advises their tentative use for the relief of dental disorders. They are not, however, to be used haphazard. It is important to bear in mind the seat of the trouble, the manner of life, the age and sex of the patient. Each remedy has its own particular sphere of action, and should be administered separately, not in combination with others, as is the practice in allopathy. In acute cases calling for prompt relief the dose should be frequently repeated,—every fifteen minutes for the first few hours,—while in chronic cases the intervals may extend to days. If after some time the symptoms become less intense, the remedy is continued, but if only some of the symptoms disappear, another medicine is indicated, just as would be the case if no change took place. When the symptoms lessen, the intervals between the doses should be increased, but the medicine should be continued until a cure is effected. The dose for women is usually less than for men. In some cases a single dose will be sufficient, but if no effect is noticed after three doses, another remedy is indicated. The action of medicine is more rapid with some persons than with others; and some remedies produce their best effect in the morning, as is the case with pulsatilla and sulphur, while others are more effective in the afternoon or evening, as nux vomica and rhuseti.

---

[1] From an article in L'Odontologia for November, 1902, by J. Cancela. Translated by Dr. B. McCullough, Philadelphia.

Medicines should be administered half an hour before meals, or one hour after, and during their administration stimulants, spices, coffee, and acids should be avoided. In selecting a remedy, the temperament of the patient is to be considered, as some remedies are better suited to one temperament than to others.

Borax, in weak solutions, he recommends internally, for the relief of apthous stomatitis. As an external application, he suggests twenty-five centigrammes of borax in fifteen grammes of pure glycerin.

Calendula is especially useful in promoting the healing of wounds and preventing suppuration. The author says, "I always use this remedy after extracting, with most beneficial results; ten grammes of the tincture in one hundred of glycerin, to paint the wounds."

Chamomile is of great value where the motor nerves are subject to excitement or are morbidly irritated. Chamomile has a special action upon the dental pulp, and is therefore of value in pulpitis, in periostitis, and in inflammatory trifacial neuralgia. In the treatment of pathological dentition, to combat the nervous irritability and consequent spasms, it has no superior. The fifth to the twelfth dilution is most generally used.

Chelidonium, used in the fifth to the twelfth dilution, has produced gratifying results in severe odontalgia, and in pain in the jaws.

Caffea is a powerful agent in difficult dentition accompanied with general nervous excitement. The third and the sixth dilution is generally preferred.

Conium is indicated in odontalgia of a throbbing character, where the seat of pain is responsive to thermal changes, and for pain caused by bridge-work or regulating appliances. For internal use the fifth to the twelfth dilution may be used, and as a lotion fifteen to twenty grammes of the tincture may be added to one-thousand grammes of pure water.

Eucalyptus is indicated as an antiseptic in dental caries, and as a tonic to the pulp. Of the different preparations, the tincture is preferred. The leaves may be chewed to perfume the breath, harden the gums, and to cure a bleeding fungous condition of the gums. Of the tincture obtained from the leaves, dilutions of from the fifth to the twelfth may be used, while for local applications cotton saturated with the tincture may be applied, protected with gutta-percha.

Gymnocladus canadensis is considered a powerful agent to combat inflammation of the tongue.

Creosotum. To the distinguished homœopathist, Dr. Teste, we owe the knowledge of this drug in its application to odontalgia. According to him it not only relieves dental pain, but retards the progress of caries, and in difficult dentition with inflammation it is of great value. From the third to the twelfth dilutions are most used.

Mercury. Its effect in increasing the secretions of the entire alimentary tract, and in increased doses causing what is known as mercurial stomatitis, suggests its use in subacute inflammations of the mouth and pharynges. Used in triturations of the third to the sixth.

Phosphorus has a characteristic action on the jaw-bones, as is seen upon workers in match-factories; therefore it is indicated in necrosis of these bones and in rickets, used in dilutions of the third to the twelfth.

MECHANICS OF THE JAWS. By Mr. J. Arbuthnot Lane, M.S., F.R.C.S.[1]

As an illustration of the comparatively serious nature of the operative interference which a condition of marked deformity may demand for its efficient treatment, I will now describe briefly the details of a case of open bite associated with all the conditions of the upper jaw which result from imperfect development of the nasopharynx, and with the enlargement of the lower jaw consequent on an excessive development of the muscles of the tongue, producing an underhung jaw and open bite. The boy was unable to bite his food, his mouth was always open, his lips were consequently thick and everted, and his general appearance was any-

---

[[1] The *Transactions of the Odontological Society of Great Britain* for January, 1903, contains a very valuable paper on "Mechanics of the Jaws," by Mr. J. Arbuthnot Lane, Surgeon, and not a dentist. An abstract is reproduced here which must prove of interest to those who have been disputing the policy of performing the operation to which he alludes. This operation, largely theoretical here has been a subject of much dispute among orthodontists and oral surgeons in this country, but it seems to have been a quite common practice with Mr. Lane. It consists of cutting away a "triangular area from the body of the lower jaw" on both sides. This operation seems to have been a universal success in this surgeon's hands without the injury anticipated by surgeons here.—ED.]

FIG. 1.

Condition of case of open bite before operation.

FIG. 2.

Condition of open bite after operation.

thing but pleasing, while his breath was offensive, and his teeth (especially the lower incisors) and gums were covered with decomposing foodstuff, etc. The state of his health was correspondingly unsatisfactory. I would just add that the amount of decomposition and inflammation that go on in the gums and about the teeth in cases of open bite, with the digestive disturbance, general depreciation, and often glandular inflammation consequent upon them, are very much greater than in ordinary mouth-breathers from nasal insufficiency alone. This is due to the fact that in the former a large number of teeth cease to perform any biting function, and tartar, etc., collect about them, while in the latter this is not the case. His mother states that as a young child his jaws and teeth were perfectly normal, and that these deforming changes appeared later in life. Fig. 1 shows the plaster casts of the jaws and teeth. In order to remedy the deformity and reduce his masticatory, respiratory, and æsthetic disability to a minimum, careful measurement of plaster casts was made, and experimental sections were made through them to determine how much bone should be removed in order to obtain the most benefit.

When this had been arrived at I cut away a triangular area from the body of the lower jaw on each side, fastening the fragments securely together with stout virgin silver wire in such a position that the front teeth fitted on those of the upper jaw as closely as possible. It was necessary to bring the anterior fragment upward into a place much above that it originally occupied to meet the receding upper incisors. This plaster cast and photograph show the present condition. As you see, the jaws are now able to perform their normal functions very satisfactorily. The wires were removed later, as they gave rise to inflammation, probably because of the very foul state of the mouth at the time of the operation. In a patient in whom I had displaced the body of the jaw forward from its normal position, I avoided risk of infection by dividing the ramus transversely at the level of the margin of the alveolus, and after bringing the body of the jaw sufficiently far forward I wired it to the ramus. This could be done in many cases of underhung or ill-developed jaw, but it was not applicable to the case I have described, because of the necessity to do away with the exceedingly deforming "open bite."

The teeth in the anterior segment of the lower jaw have been uninfluenced by the section of their nerves and blood-vessels. In every case in which I had previously divided the lower jaw on either side in order to improve the biting capacity or the appearance of the patient, I found that the teeth in the anterior segment apparently suffered no injury. Indeed, in this particular instance the condition of the teeth, as that of the mouth generally, and the health of the individual have been very greatly improved by the absence of the offensive decomposing, pasty material which covered his teeth, resulting from the functional inactivity of certain teeth and from the excessive ventilation of the mouth consequent on the high degree of open bite. Now that he can keep his mouth shut habitually there is no longer the same accumulation of desiccated epithelium and food about the teeth, and the absence of this material and of the toxins thus produced, as well as his capacity to eat his food properly, are responsible for the very marked improvement in his health. His lips have lost their thick, flaccid, everted form, and are now thin, firm, and in apposition, suggesting a higher degree of intelligence than before.

Curiously enough, the tongue has altered in form, owing to the wasting of its anterior portion, which has resulted from the diminution in the size of the bony space accommodating it. It is as broad and thick as it was before, but is very distinctly shorter, and its anterior portion is thinner and more pointed. I would remind you of a condition illustrated by this and other cases of open bite and superior protrusion, and with which you are probably thoroughly familiar,—namely, the different behavior of the upper and lower incisors where their functions are in abeyance. While the lower incisors project upward beyond their normal level and fan out above the plane of the other teeth, the upper incisors do not reach the normal level. Both form arcs with the concavity open downward. This condition existed in this boy before operation, but the restoration of the function of the incisor teeth appears to have caused the upper ones to descend somewhat, so as to occupy a better working level. Fig. 2 represents the condition of the jaws after the operation. I am indebted to Mr. Spiller for this case of open bite, etc., and for his most kind and valuable help in many difficulties. He tells me that after a few months had elapsed since the operation sensation in the teeth in the anterior segment of the mandible had been restored to the normal, showing that the continuity of the divided inferior dental nerves had been restored.

DISCUSSION.

The President thought the Society was greatly indebted to the author for the trouble he had taken in preparing such an excellent practical paper. The cases described came largely under their ken, there was no man in the room who had not seen similar cases; but he believed the removal of the V-shaped section from the lower jaw on each side was somewhat novel. He had read of such a thing in books. but had never seen the actual models of a case before. Such cases were observed in practice, and it was really very difficult to know how to treat them. He thought it must be very gratifying to the author to see such success attending his treatment of this case.

Mr. Tomes thought the paper was very difficult to discuss off-hand; to discuss it properly it was necessary to sit down and quietly read it, look at the illustrations and casts, and then think the subject well out. One casual matter had passed through his mind while listening to the paper. The boy with the syphilitic tongue was assumed—he did not mean in an antagonistic or an offensive sense—to have altered the position of his teeth. He would like to ask the author what the tongue looked like, because one's experience of syphilitic tongues was that so far from altering the position of the teeth, they themselves became conspicuously indented by the teeth. The tongue was a very soft thing, but still very soft growths, indeed, in the mouth, would displace the teeth and push them out of the way. He had seen teeth very greatly displaced by a tumor of the cheek, so soft that on touching it with the finger it felt as if it would go right through the tumor; and yet soft as it was, by constant pressure it displaced the teeth of an adult. The syphilitic tongue with which they were familiar did not displace the teeth, but became indented; he would therefore like to know what the boy's tongue was like, and whether it had the softness and flabbiness of most syphilitic tongues. In regard to the question of the imperfect development of the nasopharynx and insufficient breathing through it, he took it the author considered that such influence upon the jaws was due rather to the imperfect development and imperfect nutrition of its own parts, and that he did not endorse the view which had been sometimes put forward that there was such a thing as a question of actual alteration of air-pressure, which had some effect. It had always seemed to him that the argument in regard to air-pressure was very weak; they were told there was an alteration of air-pressure, which would have certain mechanical effects, but had never been told what that alteration of air-pressure was; they had never had submitted to them, at least not to his knowledge, measurements of alterations of air-pressure. If there was an alteration of air-pressure in the imperfect nasopharynx which did not exist in the ordinary state, it was a fact which was capable of very definite experimental demonstration, and so far as he knew that had not been done. Hitherto, assumptions of diminutions of pressure and increase of pressure had been given. He was informed by Dr. Creasy that he did not think the author endorsed that view.

Mr. F. J. Bennett said he would like to answer the question asked by Mr. Tomes, by narrating an experiment he performed when the question of negative pressure arose.

He placed an aneroid barometer in a glass bulb, connecting it with a tube open at both ends. With the aid of an ordinary bicycle pump he endeavored to exhaust the air and to ascertain whether there was any action on the aneroid barometer. There was none whatever. As soon as he closed one end of the tube the needle of the barometer swung round at least a quarter of a circle, and would have gone more if he had exhausted the air further. He concluded that it was only in the event of the mouth being closed as well as the antral cavity, that any exhaustion in the antrum could be obtained.

Mr. J. F. Colyer suggested that as it was almost impossible to discuss the paper without reading it, the discussion should be adjourned for two months. In that way the members would learn very much more from the discussion than they would if it were taken on the present occasion. Many points in the paper were open to criticism, and it would be in the interests of all concerned to put one's remarks in a proper form.

Mr. Constant seconded Mr. Colyer's suggestion that the discussion should be adjourned. He thought the subject was a very important one, and, as Mr. Colyer had pointed out, there were several points in the paper which many members would like to carefully consider and speak upon later. He had travelled over two hundred miles in order to hear the paper, and was not disappointed. The author had very clearly insisted upon the necessity for laying down more definite rules than at present existed with regard to the normal physiological development of the jaws, the forces that brought the teeth into play, and so on, and that alone was very valuable. He would like to ask the author whether he kept careful notes of cases of nasal obstruction in which there was no deformity of the teeth. He had done so during the last ten years, and should be very happy at any time to compare the results of his notes with those of the author.

It was unanimously agreed that the discussion on the paper should be adjourned to March 30.

## Figures and Tables

**Fig. 1. f1:**
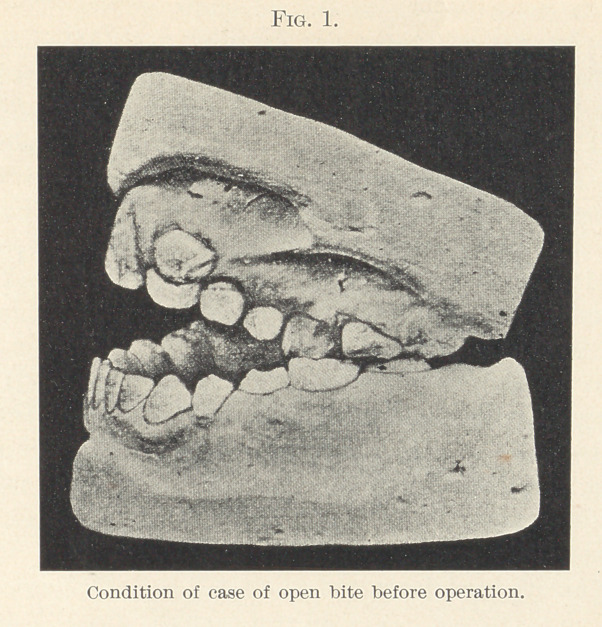


**Fig. 2. f2:**